# Mutations in *RAB39B* in individuals with intellectual disability, autism spectrum disorder, and macrocephaly

**DOI:** 10.1186/s13229-017-0175-3

**Published:** 2017-11-09

**Authors:** Marc Woodbury-Smith, Eric Deneault, Ryan K. C. Yuen, Susan Walker, Mehdi Zarrei, Giovanna Pellecchia, Jennifer L. Howe, Ny Hoang, Mohammed Uddin, Christian R. Marshall, Christina Chrysler, Ann Thompson, Peter Szatmari, Stephen W. Scherer

**Affiliations:** 1 Institute of Neuroscience, Newcastle University, c/o Sir James Spence Institute, Queen Victoria Road, Newcastle upon Tyne, NE1 4LP UK; 2 Program in Genetics and Genome Biology, The Centre for Applied Genomics, The Hospital for Sick Children, Toronto, ON Canada; 3 McLaughlin Centre, University of Toronto, Toronto, ON Canada; 4 Autism Research Unit, The Hospital for Sick Children, Toronto, ON Canada; 5 Mohammed Bin Rashid University of Medicine and Health Sciences, Dubai, UAE; 6 Department of Psychiatry and Behavioural Neurosciences, McMaster University, Hamilton, ON Canada; 7 Centre for Addiction and Mental Health, The Hospital for Sick Children and University of Toronto, Toronto, ON Canada; 8 Department of Molecular Genetics, University of Toronto, Toronto, ON Canada

**Keywords:** *RAB39B*, Intellectual disability (ID), RNAseq, Whole genome sequencing (WGS)

## Abstract

**Background:**

Autism spectrum disorder (ASD), a developmental disorder of early childhood onset, affects males four times more frequently than females, suggesting a role for the sex chromosomes. In this study, we describe a family with ASD in which a predicted pathogenic nonsense mutation in the X-chromosome gene *RAB39B* segregates with ASD phenotype.

**Methods:**

Clinical phenotyping, microarray, and whole genome sequencing (WGS) were performed on the five members of this family. Maternal and female sibling X inactivation ratio was calculated, and phase was investigated. Mutant-induced pluripotent stem cells engineered for an exon 2 nonsense mutation were generated and differentiated into cortical neurons for expression and pathway analyses.

**Results:**

Two males with an inherited *RAB39B* mutation both presented with macrocephaly, intellectual disability (ID), and ASD. Their female sibling with the same mutation presented with ID and a broad autism phenotype. In contrast, their transmitting mother has no neurodevelopmental diagnosis. Our investigation of phase indicated maternal preferential inactivation of the mutated allele, with no such bias observed in the female sibling. We offer the explanation that this bias in X inactivation may explain the absence of a neurocognitive phenotype in the mother. Our cellular knockout model of *RAB39B* revealed an impact on expression in differentiated neurons for several genes implicated in brain development and function, supported by our pathway enrichment analysis.

**Conclusions:**

Penetrance for ASD is high among males but more variable among females with *RAB39B* mutations. A critical role for this gene in brain development and function is demonstrated.

**Electronic supplementary material:**

The online version of this article (10.1186/s13229-017-0175-3) contains supplementary material, which is available to authorized users.

## Background

Genomic microarray technology and genome sequencing are changing our understanding of the genetic architecture of autism spectrum disorder (ASD). This lifelong neurodevelopmental disorder of early childhood onset [1] is now known to be associated with rare inherited and de novo genetic variation, comprising single nucleotide variants (SNV), smaller and larger insertions and deletions (indels and copy number variants, CNVs, respectively), and other complex structural variation [2–4]. ASD affects males on average four times more frequently than females, which strongly suggests an etiological role for the sex chromosomes. However, the majority of genes implicated in ASD, so far, are autosomal, with notable exceptions including *MeCP2*, the *NLGN* genes, and *FMR1*. With an estimated 400 or more genes involved in ASD’s etiology [5], there may remain several undiscovered ASD susceptibility genes on the sex chromosome. For example, some X-linked genes involved in intellectual disability (ID) may be contributory factors in ASD, particularly as more than 100 genes on the X chromosome have now been described in association with ID [6].

Our own whole genome sequencing (WGS) study (www.mss.ng), currently comprising 2620 ASD genomes from 2066 unique families, has identified a number of X chromosome genes in which rare predicted damaging mutations are enriched, including *MECP2*, *NLGN3*, *NLGN4*, and *PCDH11X* [7]. One such gene, *RAB39B*, has been described in the literature in association with X-linked ID along with variable phenotypic manifestations including ASD, seizures, macrocephaly, delayed psychomotor development, and early-onset Parkinson’s disease or Parkinsonism [8–11]. This two-exon gene, which comprises two protein domains, is a member of the RAS oncogene family with known expression in neurons and neuronal precursors, particularly in the hippocampus [8]. Along with other RAS proteins, it plays a key role in intracellular vesicular trafficking. Specifically, through its trafficking role, *RAB39B* is now known to mediate the surface expression of GluA2, a subunit of the glutamate AMPA receptor [12].

Here, we present a detailed description of a family in which two brothers with ASD have a maternally inherited loss of function (LoF) mutation in exon 2 of *RAB39B*. Both males are now in their early 30s and have ID with no functional language; they also have fine motor difficulties and macrocephaly. Additionally, one presents with a childhood onset unilateral hand tremor and has marked slowing in the execution of simple, routinized movements. In addition, their female sibling, who shares the *RAB39B* mutation, presents with mild ID and a broad autism phenotype (BAP), but not ASD. Finally, the transmitting mother has a unilateral fine upper intention tremor of unknown etiology. We examined the impact of an exon 2 mutation on gene expression in differentiated neurons using CRISPR/Cas.

## Methods

The family described was recruited as part of ongoing studies investigating the genetic etiology of ASD [7]. ASD diagnoses are made by expert clinicians using the Autism Diagnostic Interview (ADI) [13] and the Autism Diagnostic Observation Schedule (ADOS) [14] combined with clinical judgment. All data were collected following informed consent from participants or substitute decision-makers, and the study was conducted with approval from the local research ethics board. The mother has provided specific written consent for this case report.

### Phenotypes

**Fig. 1:**
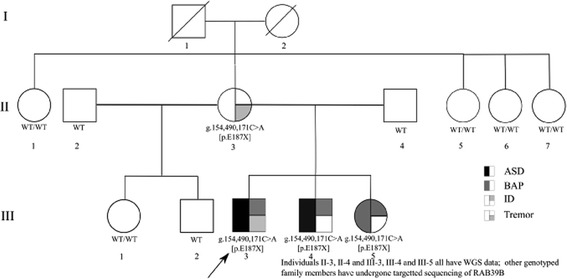
Annotated pedigree of family described in the text

Both affected males were recruited into the study at the same time, one aged 9 years (III-3, hereafter “proband”) and the other 8 years (III-4) (Fig. 1). They had both been diagnosed with ASD at age 3 years, and on recruitment into the study, diagnostic clarification was sought with ADI-R and ADOS-G. Additionally, nonverbal IQ was measured using the Leiter-R, language using the Oral and Written Language Scales (OWLS-II), and adaptive function using the Vineland Adaptive Behavior Scales-II (VABS). Their female sibling (III-5) was first assessed in a specialist pediatric clinic because of developmental concerns age 5 years. At the time of recruitment (age 6 years), she completed a measure of IQ (Wechsler Intelligence Scale for Children) and her adaptive function was measured using the VABS. Assessment for ASD was not completed during her childhood, but age 29 years the presence of autism traits of childhood onset were ascertained by a maternally completed Social Responsiveness Scale (SRS) [15]. Head circumference was also collected, and craniofacial features were evaluated. Medical history was collected by way of a detailed questionnaire. The two males with ASD were re-evaluated age 33 years (III-3) and 32 years (III-4). The Leiter-R and VABS were both repeated, and interim medical history was collected. Both parents completed the Toronto Alexithymia Scale [16]. This scale measures alexithymia, characterized by an inability to self-reflect on and label emotions. Although neuropsychiatric and developmental history was not specifically sought for either parent, both are professionally employed and neither endorsed an ASD diagnosis, or broader phenotype, during the collection of family history.

### Genotypes

Members of the family were genotyped using either an Affymetrix 500k (II-3, II-4, and III-3) [17] and/or Illumina 1M (II-3, II-4, and III-4) [18, 19] microarray, and CNVs were detected as previously published [18–20]. Briefly, multiple different CNV calling algorithms were used to generate high-confidence CNV calls, with support from a minimum of two algorithms required, and CNVs with a frequency of < 0.1% in controls (*N* = 9611) prioritized as “rare.” CNVs with > 50% reciprocal overlap were deemed identical. We also removed all CNVs that had > 50% overlap with a known segmental duplication. No CNVs of clinical significance, or of uncertain clinical significance, were identified in this family. The proband, his male and female siblings, and both parents also underwent whole genome sequencing (WGS) by Complete Genomics (Mountain View, CA) as previously published [21]. Identified variants were annotated and likely deleterious mutations prioritized to capture those that were rare (MAF ≤ 1%), LoF (nonsense, splice site, and frameshift), and damaging de novo missense mutations (damaging as evidenced by SIFT ≤ 0.05, Polyphen2 ≥ 0.95, CADD ≥ 15, Mutation Assessor score ≥ 2 and PhyloP ≥ 2.4) [21]. For filtering, MAF was based on the following control samples: 1000 Genomes Project, NHLBI-ESP, ExAC, and Complete Genomics whole genome sequencing control data (CGI diversity panel and Wellderly samples). Variants reported in this paper have been validated by Sanger sequencing.

### Extended family

Several members of the extended family (Fig. 1) were genotyped for the *RAB39B* mutation. Although these individuals were unavailable for neuropsychiatric evaluation, phenotypic data was collected through a family history review provided by the mother (II-3).

### X inactivation

The X inactivation (XI) ratio was calculated by characterizing genotyped alleles according to the number of CAG repeats on the highly polymorphic androgen receptor at Xq11-q12. The methylation-sensitive restriction enzymes Hpa II and Hha I were used to cut the repeat region on the active (but not inactive) strand before and after quantitative PCR to determine the X inactivation ratio. Non-methylated (active X) DNA segments digest with the enzymes and are thereby unavailable for PCR amplification. Methylated (inactive X) Hpa II/Hha I sites do not digest with enzyme and remain intact for amplification. Post-digestion PCR products therefore represent methylated (inactive X) DNA sequences only. The androgen receptor was also genotyped on paternal and male offspring X-chromosomes to facilitate phasing of the mutated allele. This thereby allowed us to determine whether the mutated or non-mutated allele was preferentially inactivated in mother (II-3) and daughter (III-5).

### Investigation of familial segregation

We estimated likely causality for the family’s *RAB39B* mutation, and for all variants in *RAB39B* described in the literature, using the method of Thompson et al. [22, 23]. This considers the segregation of the variant across the full pedigree from which a Bayes factor (BF) is calculated. As articulated in the new ACMG-AMP guidelines for variant classification [24], a BF of 8 or more provides “supportive” evidence of pathogenicity, of 16 or more “moderate” evidence of pathogenicity, and of 32 or more “strong” evidence of pathogenicity.

### iPS culture and differentiation

Skin fibroblasts from a healthy man were obtained at the Hospital for Sick Children under the approval of the SickKids Research Ethics Board, and induced pluripotent stem cells (iPSCs) were generated and characterized as previously published [25]. Cultured iPSCs were maintained on Matrigel® (Corning). One million iPSCs were nucleofected using Nucleofector II (Amaxa) device with program A-023 in Solution I with 5 μg Cas9D10A plasmid (Addgene, #44720), 3 μg each of two gRNA cloning plasmids (Addgene, #41824) containing paired gRNAs A (AAGAGGTTGTCAAATCAGAGAGG) and B (TCTGAAGAGTGAACCACATTTGG) targeting exon 2 of *RAB39B*, and 1 μl of 100 μM single-stranded oligonucleotide (ssODN) containing two 60 nucleotide-long arms of homology flanking a 3xSTOP, i.e., stop codons in all three reading frames. This introduced mutation is not the same as that involved in the family described. Cells were evenly distributed in a 96-well plate. At confluency, genomic DNA was extracted and droplet digital PCR (ddPCR) was performed to determine the absolute quantification of mutant alleles in each well. The mutant stem cell clone was then differentiated into cortical excitatory neurons. A comparison cell line was generated from the isogenic control line, i.e., the same line from which the knockout described above was made.

### RNAseq

RNeasy® mini kit (Qiagen) was used to extract total RNA from mature neurons. RNA samples were submitted to an Agilent Bioanalyzer 2100 RNA Nano chip for quality control. Concentration was determined using Qubit RNA HS Assay on a Qubit fluorometer (ThermoFisher). RNA libraries were prepared using NEBNext Ultra RNA Library Preparation kit for Illumina. Briefly, 500 ng of total RNA was used for poly-A mRNA enrichment before being split into 200–300 bp fragments for 4 min at 94 °C. Fragments were converted to double-stranded cDNA, end-repaired, and adenylated in 3′ to create an overhang A, allowing ligation of Illumina adapters with an overhang T. Fragments were amplified and RNA library integrity was verified on a Bioanalyzer 2100 DNA high-sensitivity chip (Agilent Technologies) and quantified using Kapa Library Quantification Illumina/ABI Prism Kit protocol (KAPA Biosystems). Stranded libraries were pooled in equimolar amounts and sequenced on an Illumina HiSeq 2500 platform using a high-throughput run mode flowcell and the V4 sequencing chemistry to generate paired-end reads of 126-bases in length, following Illumina’s recommended protocol. Data quality was assessed using FastQC v.0.11.2. Trimmed reads were screened for presence of rRNA and mtRNA sequences using FastQ-Screen v.0.4.3. RSeQC package v.2.3.7 was used to assess read distribution and positional read duplication and confirm strandedness of alignments. Raw trimmed reads were aligned to the reference genome hg19 using Tophat v.2.0.11. Tophat alignments were processed to extract raw read counts for genes using htseq-count v.0.6.1p2. Raw gene counts were loaded and sample-normalized using DESeq v.1.18.0. Principal component analysis (PCA) was performed to assess relation among samples. Two-condition differential expression was done with the edgeR R package, v.3.8.6 on four (*RAB39B* knockout) and six (control) independent experiments. Cpm filter was adjusted to the size of each library set to obtain at least 8–10 reads in at least two samples. EdgeR results were evaluated using FDR with a cutoff of 0.05.

#### Pathway enrichment

Pathway enrichment analysis was then performed using the R package goseq version 1.24.0 and R version 3.3.1 (2016-06-21) using a custom gene set collection including 6008 from gene ontology (GO, obtained from the R package GO.db version 3.3.0) and 1723 from pathways (KEGG, Reactome, NCI collections downloaded from the respective websites on 20170120). GO terms were obtained from the R package org.Hs.eg.db v3.4.0. Gene sets were filtered by size to remove both very generic and very specific terms. To aid in reproducibility, the filtered gene set definitions are in Additional file 1: human_GO_15_800_PATH_10_500.gmt.

## Results

### Phenotypes

The proband (III-3, Fig. 1) was evaluated age 3 years because of parental concerns regarding speech delay and poor socialization and diagnosed with ASD. Parents reported that his speech was developing well up until 18 months with the acquisition of a small number of well-articulated words, but then plateaued. At 36 months, he presented as a loner who had no interest in communicating or interacting with others, and he failed to use eye-to-eye contact. Gross and fine motor milestones developed within normal limits, and there were no other medical concerns. Morphological abnormalities included a head circumference on the 98th percentile and finger syndactyly. By 8 years, his language had improved, although was comprised predominantly of echolalia. Assessment of intellectual ability and adaptive functioning age 9 years were consistent with a diagnosis of ID, and ADI-R [13] and ADOS [14] assessments completed at this time were both consistent with a diagnosis of ASD (Table 1 and Additional file 2: Table S1). He was further assessed age 33 years. Interim medical history was negative for major medical or psychiatric illness, and re-evaluation at this time revealed very little change in symptoms. A number of movement-related symptoms were noted, including impaired fine motor control and episodic difficulties initiating movements. He was also noted to have a fine bilateral tremor of his hands, worse when agitated or anxious.

**Table 1:** Genetic and clinical data from family segregating a *RAB39B* mutation

	II-3	II-4	III-3	III-4	III-5
Age at first assessment (months)	NA	NA	36	36	60
Sex	F	M	M	M	F
Microarray^a^	**NS**	**NS**	**NS**	**NS**	**NS**
WGS^b^	Xq28, *RAB39B*, g.154,490,171C>A 3p26.3, *CNTN6*, g.1,443,180G>A	WT *RAB39B* WT *CNTN6*	Xq28, *RAB39B*, g.154,490,171C>A 3p26.3, *CNTN6*, g.1,443,180G>A	Xq28, *RAB39B*, g.154,490,171C>A 3p26.3, *CNTN6* g.1,443,180G>A	Xq28, *RAB39B,* g.154,490,171C>A WT *CNTN6*
Growth					
Head circumference (%ile)	NA	NA	98th	98th	50th
Neurodevelopment					
Full-scale IQ	110	109	39	32	69
Speech delay	NA	NA	+	+	+
ASD	−	−	+	+	+
Other neurodevelopmental	NA	NA	−	−	−
Neurological					
Epilepsy	−	−	−	−	−
Parkinson	−	−	−	−	−
Other neurological	Tremor	NA	Tremor, bradykinesia, poor fine motor	Poor fine motor	−
Congenital	NA	NA	Syndactyly	Syndactyly, epicanthus, high-arched palate	Left epicanthus
Other medical	NA	NA	−	−	−

*NA* information not available, *+/−* positive/negative for attribute

^a^ *NS* implies no variants of pathological significance, or variants of unknown significance contributing to ASD identified

^b^ *WT* wild type

The proband’s male sibling (III-4) was evaluated age 3 years because of parental concerns with language delay and diagnosed with ASD. At 36 months, he used no functional language and was socially aloof. His motor milestones were normal. He was on the 98th percentile for head circumference and had epicanthic folds, a high-arched palate, and a finger syndactyly. By 6 years, he had developed some language skills, although failed to use this to facilitate interaction with others. Assessment of intellectual ability and adaptive functioning age 8 years were consistent with a diagnosis of ID, and ADI-R and ADOS at the time were both consistent with ASD (Table 1). When re-evaluated age 32 years, he had some limited language skills although his nonverbal IQ and adaptive skills had changed little from childhood. An interim medical history did not identify any major medical or psychiatric illness. No movement-related symptoms were identified, although he, too, had major difficulties with fine motor control, impacting feeding, dressing, and other instrumental activities of daily living.

Their younger female sibling (III-5) was first evaluated when she was 5 years because of concerns with speech delay. She was described as responsive and curious as an infant and became securely attached to her primary caregivers. However, her speech developed late, and articulation and intonation abnormalities were noted. She was described as aloof from her peers and content to play by herself. Assessment of intellectual ability and adaptive functioning age 6 years were consistent with a diagnosis of ID (Table 1). Morphologically, she was on the 50th percentile for head circumference at the time of assessment and had a left-sided epicanthus. Although she was not assessed for ASD during childhood, a maternally completed SRS [15] when she was 29 years indicated a moderate degree of impairment, consistent with BAP. She was unavailable for further diagnostic testing.

Both parents completed a range of assessments that indicated intellectual ability and language functioning in the average range. Both parents’ scores on the Toronto Alexithymia Scale [16] were consistent with alexithymia (father = 70, mother = 60, scores 61 and above being consistent with alexithymia). At the time of assessment, parents were in full-time professional employment. Maternal self-report indicated a diagnosis of a persistent left-sided intention tremor of unknown etiology, but no other neurological history. Members of the extended family were unavailable for phenotypic evaluation (Fig. 1). However, review of the family history according to the mother was negative for ASD, other developmental disorders, neuropsychiatric diagnoses, or major medical illness in extended family members.

### Genetics

A nonsense variant in exon 2 of *RAB39B* (Xq28, g.154,490,171C>A, c.559G>T, [p.E187X], NM_171998, CADD=12.46) was identified in both males with ASD, inherited from their mother. The female sibling was found to be heterozygous for this variant by WGS and Sanger sequencing. The variant was absent from all members of the extended family who were tested (Fig. 1). Analysis of X inactivation in the mother and daughter showed the mother had preferential inactivation of the mutated allele (67%:33%, mutated allele: non-mutated allele), whereas her daughter showed no such bias (48%:52%, mutated allele: non-mutated alleles). *RAB39B* is given a pLI score of 0.72 in the Exome Aggregation Consortium (ExAC), consistent with intolerance to mutation [26]. No individuals with this mutation are listed in ExAC. A Bayes factor calculated from the pattern of segregation of this mutation in the family is consistent with moderate to strong evidence of pathogenicity according to the ACMG-AMP guidelines (BF = 26.1) [24].

The two affected males also inherited from their mother a nonsense variant in *CNTN6* (3p26.3, g.1,443,180G>A [p.W923X], NM_001289080, CADD=13.85), a gene implicated in ASD. This variant is observed in one non-Finnish European individual in ExAC, which assigns this gene a pLI score of 0, suggesting greater tolerance to mutation than *RAB39B*. The frequency of this mutation in ExAC is 8.44e-06. This mutation was not shared by their female sibling. However, one maternal sibling reported to be normal from a neurodevelopmental perspective also had this mutation (II-7). No clinically significant CNVs were identified.

We investigated our own data for evidence of *RAB39B* mutations in other individuals with ASD (*N* = 2620) (www.mss.ng). One male was identified with a maternally inherited 13 kb deletion (chrX:154,477,501-154,490,661 [hg19]). He is described as exhibiting classic symptoms of ASD with speech delay (age of single words = 78 months). No further information is available. We also investigated for the presence of CNVs and SNVs in *CNTN6*. Among cases with ASD, five individuals have exon impacting SNVs and 15 exon impacting CNVs (3 deletions and 12 duplications). No individuals were identified with variants impacting both *CNTN6* and *RAB39B*.

### iPSC and expression studies

**Fig. 2:**
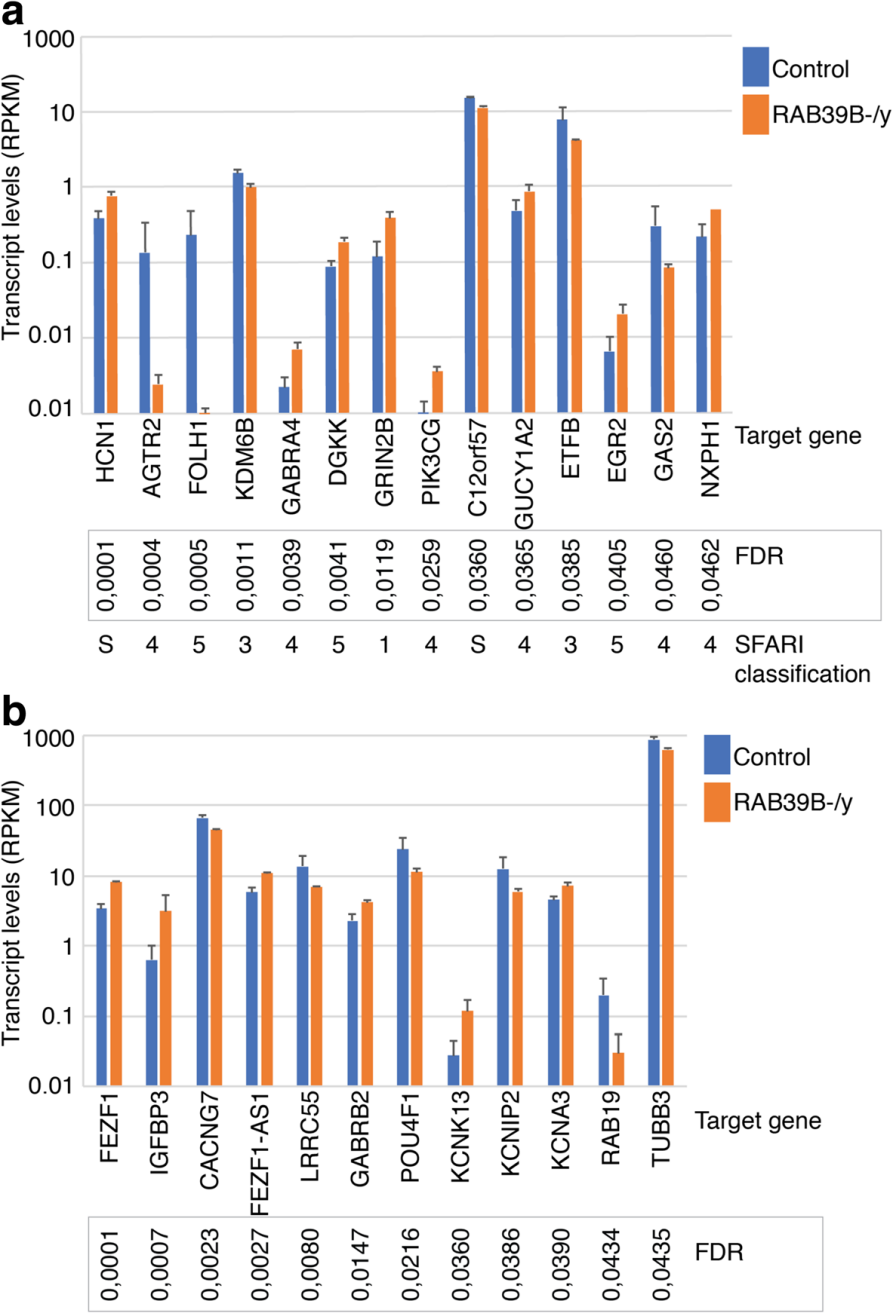
Levels of transcript of selected genes in control and RAB39B-/γ-glutaminergic neurons as determined by RNAseq experiment. Values are presented as mean ± SD of four (RAB39B) and six (control) independent experiments

We used CRISPR/Cas to create a mutation in exon 2 of *RAB39B*. Although the mutation generated in this way is not identical to the family’s, it does provide insight into the impact of an exon 2 loss of function mutation on differential patterns of gene expression. A number of genes were differentially expressed between mutant and control lines (Fig. 2a, b; Additional file 3: RAB39B4_vs_Controls6_RPKM_Filt.txt). Figure 2 specifically highlights those differentially expressed genes that were previously found associated with ASD and are present in the Simons Foundation Autism Research Initiative (SFARI) database. Among these top hits are known SFARI syndromic genes (*HCN1*, *C12orf57*) and one high confidence ASD-risk gene (*GRIN2B*). There are also two genes, *GABRA4* and *GABRB2*, that code for subunits of the GABA receptor, the major inhibitory neurotransmission unit in the mammalian brain. Moreover, several genes encode elements of the potassium channel (*KCNIP2*, *KCNK13*, *KCNIP2*). Finally, *NXPH1* encodes a protein that forms complexes with alpha-neurexins, implicating its role in cellular adhesion in the brain. We next conducted gene set enrichment analysis to identify the associated GO terms with the 260 differentially expressed genes with FDR < 0.05 (Additional file 4: enrichment_RAB39B4_vs_Controls6.txt). Only downregulated genes were significant, with their GO terms provided in the Additional file 5: Table S2. The enriched terms point to the involvement of these downregulated genes in neuron fate, neurogenesis, and regulation of nervous system development.

**Fig. 3:**
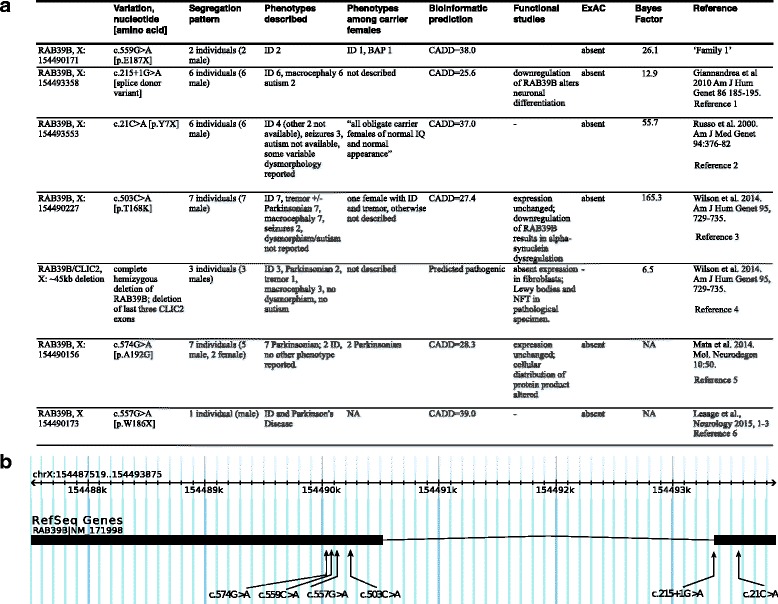
Literature curation for RAB39B mutations

We curated all of the descriptions of *RAB39B* patients from the literature (Fig. 3). Five families have been described, each with a unique variant [8, 9, 11], along with one further individual identified from a sample of 1348 patients with Parkinson’s disease (PD) [10]. In one family (Fig. 3, Reference 1), six males with ID and macrocephaly are described with a *RAB39B* splice donor variant (hg19: 154,493,358, c.215+1G>A) [8]. Two males are also diagnosed with ASD. In another family (Fig. 3, Reference 2), four males with ID, three of whom were also diagnosed with epilepsy, inherited a *RAB39B* nonsense mutation in exon 1 (hg19: 154,493,553, c.21C>A [p.Tyr7X]) [8]. In two other families (Fig. 3, References 3 and 4), macrocephaly, ID, and Parkinsonian symptoms are reported [9]. In both families (7 males and 3 males respectively), all males exhibit ID and, additionally, Parkinsonian symptoms, ranging from childhood onset tremor to akinetic-rigid PD of early onset and characterized by Lewy body brain pathology. The underlying mutation in one family is a missense mutation (hg19: g.154,490,227, c.503C>A [p.Thr168Lys]), bioinformatically predicted to be damaging, while the other family has a 45 kb deletion spanning the whole *RAB39B* gene along with the distal 3 exons of the adjacent gene *CLIC2*, also involved in X-linked ID. One other family comprises seven males with Parkinsonian symptoms (Fig. 3, Reference 5), two of whom were diagnosed with ID. This family has a missense variant (hg19: g.154,490,156, c.574G>A [p.A192G]). One further male individual (Fig. 3, Reference 6) identified in a clinical sample of 1348 individuals with PD had a nonsense mutation and presented with ID and PD, but no further family history is available. Five unique point mutations are described in these patients, with two residing in the C-terminal chain (one missense, one nonsense), both in families ascertained for PD but with incomplete penetrance for ID. CADD scores for all point mutations are consistent with pathogenicity, and none are reported in ExAC (Additional file 5). Moreover, calculations of co-segregation for each family were consistent with strong evidence of pathogenicity in 2, supporting evidence in 1 but only marginal odds in favor of pathogenicity in the other (Fig. 3).

## Discussion

This paper illustrates the clinical features in a family segregating a nonsense mutation in *RAB39B*. Consistent with previously described cases, both affected males had ID with macrocephaly [8]. In addition to ID, both males had language delay and fine motor deficits and were diagnosed with severe ASD. Despite the association with ASD demonstrated by these two cases, examination of our wider ASD datasets (microarray and WGS) found only one further individual with a variant in *RAB39B*. We also expand the phenotype to include musculoskeletal abnormality, specifically syndactyly, which was observed in both affected males. We had the opportunity to reassess the brothers in their early 30s. Although the literature also describes early-onset PD among individuals with *RAB39B* mutations [9–11], neither individual’s presentation was consistent with PD, although one male presented with a tremor and bradykinesia. It remains possible, of course, that PD may develop later in these individuals. Additionally, no change in severity of ASD symptomatology was observed into adulthood, and both males remained dependent on others for all aspects of care.

Our calculation of co-segregation was consistent with moderate evidence of pathogenicity. Its absence in ExAC and the calculated pLI score of 0.72 are consistent with intolerance of this gene to mutation. The other families described in the literature are also characterized by strong co-segregation of the mutation with phenotype, with odds (BF) in favor of causality varying from 6 to 165. Based on the evidence, therefore, it seems reasonable to conclude at this stage that rare damaging missense and LoF mutations in *RAB39B* are highly penetrant for neurodevelopmental phenotypes among males.

Our study also examined the clinical characteristics in the unaffected female sibling. This individual presented with milder developmental vulnerabilities than her siblings, although she clinically has ID and BAP. Also of note, the transmitting mother in our family had developed a fine motor tremor of her left upper limb, diagnosed by her family physician as an intention tremor of uncertain etiology. It is unclear whether this is related to her *RAB39B* mutation. The DECIPHER [27] and ISCA [28] databases contain descriptions of female carriers of *RAB39B* CNVs who have developmental phenotypes including ID, but none of those described has a movement disorder. In general terms, the *RAB39B* families described in the literature do not include phenotypic descriptions for the carrier females: several allude to normal phenotype and only one specifically describes a female carrier with ID and tremor.

Our own data at least support the strong possibility that some female carriers of *RAB39B* mutations are not without clinical consequence, although the mechanism is unclear. Phenotypic manifestation among female carriers of X-linked disorders has been previously reported, and, with the notable exception of X-linked dominant disorders such as Rett syndrome, are deemed to be due to skewed patterns of XI [29]. Specifically, skewed inactivation, which results in the normal allele being inactivated significantly more frequently than the mutated allele, may result in clinical consequence. Our own investigation of XI in this family identified preferential inactivation of the mutated allele in the mother, with no such bias in her daughter. This differential pattern of XI may offer an explanation for the absence of neurocognitive phenotype in the mother, but presence of broad autism phenotype in her daughter.

Both male offspring also harbored a *CNTN6* loss of function mutation, which was maternally inherited and shared by a maternal aunt. This gene belongs to the contactin family of proteins, a group of brain-expressed proteins that mediate cell surface interactions during nervous system development and are key proteins in the development of sensory-motor pathways. A recent study has identified that, while not being fully penetrant, a significant enrichment is observed among individuals with ASD for deletions and private coding sequence variants in *CNTN6*, with a phenotype that also includes hyperacusis [30]. We are unable to examine the relative contributions of mutations in *CNTN6* and *RAB39B* in this family, and given the low frequency and variable location of mutations in these genes, currently available datasets are unlikely to offer much more in the way of statistical power. Indeed, only five other individuals were identified with SNVs impacting *CNTN6* and 15 with CNVs, among whom only three were deletions. In our own experience, this situation is not unusual however, perhaps suggesting a modifying impact of one of the genes on phenotype [31].

Recent research has begun to elaborate the function of *RAB39B*. It is strongly brain-expressed, the expressed protein principally located in the Golgi apparatus of neurons. It has been shown to interact with PICK1, with RAB39B-PICK1 interaction a necessary step in RAB39B mediated trafficking of GluA2 [12], thereby altering synapse activity. We extend these molecular findings through our own knockout of exon 2 of *RAB39B* using CRISPR/Cas in conjunction with iPSCs. Differential expression analysis identified a number of key brain-expressed genes. A number, such as *GRIN2B*, *GABRA4*, *GABRB2*, and *NXPH1*, were upregulated, whereas others, including *CACNG7* and *ETFB*, were downregulated. Several of these have already been earmarked for their crucial role in neurodevelopment, supported by our GO enrichment analysis. In particular, the two enriched terms “regulation of nervous system development” and “regulation of neurogenesis” are consistent with *RAB39B*’s wider role in multiple brain processes.

## Conclusion

In summary, we have described a family segregating a nonsense mutation of *RAB39B* occurring in association with a variable phenotype including ID, ASD, and motor symptoms. Our study extends the *RAB39B* literature in the observation that female carriers are not without clinical consequence, with the two female carriers of this X-linked mutation in our family exhibiting milder clinical features. Newly identified cases with mutations of this gene will need to be followed through early adulthood and beyond to fully evaluate their motor function and for evidence of neurodegenerative features and, in this way, to fully grasp the spectrum of phenotype associated with aberrations of this gene.

## Additional files

Additional file 1: human_GO_15_800_PATH_10_500.gmt (filtered gene-set definitions) (GMT 4425 kb)

Additional file 2: Additional phenotype information (DOCX 122 kb)

Additional file 3: RAB39B4_vs_Controls6_RPKM_Filt.txt (differential expression analysis results) (TXT 4721 kb)

Additional file 4: enrichment_RAB39B4_vs_Controls6.txt (GO terms associated with differentially expressed genes) (TXT 484 kb)

Additional file 5: Table S2 Enriched GO terms for the 260 differentially expressed genes on the RAB39B exon 2 knockout (TIFF 262 kb)

## Abbreviations

ASD: Autism spectrum disorder
BAP: Broad autism phenotype
CNV: Copy number variant
ID: Intellectual disability
SNV: Single nucleotide variant
XI: X inactivation
WGS: Whole genome sequencing
