# Supplementary material for: Mutations in RAB39B in individuals with intellectual disability, autism spectrum disorder, and macrocephaly
Source: Mol Autism. 2017 Nov 9;8:59. doi: 10.1186/s13229-017-0175-3 (PMC5679329; doi:10.1186/s13229-017-0175-3)
Supplement: Supplementary file 2 — Additional phenotype information (DOCX 122 kb) [file 13229_2017_175_MOESM2_ESM.docx]

| ID (see pedigree) | III-3 | III-4 |
| --- | --- | --- |
| Age at testing | 33 years | 31 years |
| Leiter-R | Composite Score=38  Extremely Low Range (<0.1 percentile) | Composite Score=36  Extremely Low Range (<0.1 percentile) |
| Oral and Written Language Scales (OWLS) | Listening Comprehension Score Standard Score=40, Extremely Low Range (<0.1 percentile), AE=2-8  Oral Expression Standard Score=40, Extremely Low Range (<0.1 percentile), AE=2-11;  Oral Language Composite Score Standard Score=40, Extremely Low Range (<0.1 percentile) | Listening Comprehension Score Standard Score=40, Extremely Low Range (<0.1 percentile), AE=2-9;  Oral Expression Standard Score=40, Extremely Low Range (<0.1 percentile), AE=2-10;  Oral Language Composite Score Standard Score=40, Extremely Low Range (<0.1 percentile) |
| Vineland Adaptive Behavior Scales (VABS) | Communication: SS=21, Low Range, <1 percentile  Daily Living Skills SS=21, Low Range, <1 percentile  Socialization: SS=20, Low Range, <1 percentile  Adaptive Behaviour Composite: SS=20, Low Range, <1 percentile | Communication: SS=21, Low Range, <1 percentile  Daily Living Skills SS=21, Low Range, <1 percentile  Socialization: SS=20, Low Range, <1 percentile  Adaptive Behaviour Composite: SS=20, Low Range, <1 percentile |

SS=standardized score; AE=age equivalent

Supplementary Table 1: additional phenotype information (see text)
